# PerHeFed: A general framework of personalized federated learning for heterogeneous convolutional neural networks

**DOI:** 10.1007/s11280-022-01119-x

**Published:** 2022-12-12

**Authors:** Le Ma, YuYing Liao, Bin Zhou, Wen Xi

**Affiliations:** 1 Xi’an Institute of High Technology, Xi’An, China; 2 National University of Defense Technology, Changsha, China

**Keywords:** Personalized federated learning, Heterogeneous model, IoT

## Abstract

In conventional federated learning, each device is restricted to train a network model of the same structure. This greatly hinders the application of federated learning where the data and devices are quite heterogeneous because of their different hardware equipment and communication networks. At the same time, existing studies have shown that transmitting all of the model parameters not only has heavy communication costs, but also increases risk of privacy leakage. We propose a general framework for personalized federated learning (PerHeFed), which enables the devices to design their local model structures autonomously and share sub-models without structural restrictions. In PerHeFed, a simple-but-effective mapping relation and a novel personalized sub-model aggregation method are proposed for heterogeneous sub-models to be aggregated. By dividing the aggregations into two primitive types (i.e., inter-layer and intra-layer), PerHeFed is applicable to any combination of heterogeneous convolutional neural networks, and we believe that this can satisfy the personalized requirements of heterogeneous models. Experiments show that, compared to the state-of-the-art method (e.g., FLOP), in non-IID data sets our method compress ≈ 50*%* of the shared sub-model parameters with only a 4.38% drop in accuracy on SVHN dataset and on CIFAR-10, PerHeFed even achieves a 0.3% improvement in accuracy. To the best of our knowledge, our work is the first general personalized federated learning framework for heterogeneous convolutional networks, even cross different networks, addressing model structure unity in conventional federated learning.

## Introduction

Federated learning (FL) is an emerging technology of artificial intelligence [1–3]. FL allows multiple clients (or devices) to collaboratively train a shared global model without exposing data from local devices. The central server coordinates multiple rounds of FL processes to obtain the final global model. FL has been applied in some scenarios (such as the next-word prediction of the input method [4]). It solves the problem of data aggregation and makes it possible to design and train some cross-institutional and cross-departmental deep learning models. In particular, for deep learning applications in IoT devices such as smart phones, FL has shown favorable performance and robustness [5]. In addition, for some clients who do not have enough private data to develop accurate local models, FL is an optional way to greatly improve the performance of their models.

However, conventional FL focuses on achieving a global model by learning common knowledge of participating devices, it fails to capture the personal knowledge for each client. Learning a single shared global model across all clients may perform arbitrarily poorly when the data distribution varies significantly across clients. In particular, a widely accepted assumption of FL is that it requires all participating devices to agree on the structure of the training model, which is normally difficult to be satisfied in many real-world applications. Secondly, previous studies have shown that the exchanged model parameters in FL processes also have the risk of privacy disclosure, which is vulnerable to model inference attacks [6–8]. Under this premise, whether to train networks with the same structure and transmit the complete model information should be reconsidered when developing practical applications. Personalized Federated Learning (PFL) was proposed recently to meet the above requirements by jointly learning a personalized model for each client [9–11]. Before diving into the detail discussion, we first illustrate how the personalized requirements mentioned in this paper affect local models.

**Motivation:** **Personalization Enforces Model Heterogeneity.** An important step in advancing the application of FL in reality is to address the personalized requirements. We elaborate the need for heterogeneous models from data, system and strategy perspectives:

- **(i)** **Personalized data:** The ultimate goal of training models is to learn the features hidden in clients’ data, and the uniform global model in conventional FL often fails to fit each participant’s local data [12]. In applications such as next-word prediction, each person has their own preferred words, and the words recommended by the global model may fit the vast majority of people, but for people with specific input habits, the recommendations may fail to provide the words they expected.
- **(ii)** **Personalized hardware resources:** Personalized hardware resources usually refers to different computing powers, network connections or battery among participants, which makes it difficult to train models with the same structure. Conducting FL on these clients with heterogeneous hardware systems would lead to a compromise of clients with high computational power to train shallower networks, resulting in a waste of resources. Therefore, heterogeneous models become a key requirement to enable the application of FL in heterogeneous system scenarios.
- **(iii)** **Personalized strategies:** In this paper, model security or other personalized requirements are categorized as local strategies, and these strategies also generate the need for model heterogeneity. Here are two realistic cases:

1. Participants are reluctant to share/receive the complete model parameters due to security concerns about shared model which may reveal their privacy [13] or be embedded with backdoors [14, 15].
2. For applications like epidemic prediction [16, 17] that require lifelong learning [18], it is not practical to retrain the model every time some events occur. For example, hospitals want to jointly learn from each other’s local COVID-19 data to further optimize their local epidemic prediction models.

These above personalized requirements enforce model heterogeneity in diverse ways. FL can be conducted only if the personalized requirements are satisfied: jointly training and interacting with heterogeneous models, and that is what we are working on.

Model heterogeneity can be divided into (i) inter-layer structural heterogeneity means models contain different numbers of convolutional layers, or even different convolutional neural networks (CNN) models; and (ii) intra-layer structural heterogeneity means the parameter matrices of the mapping convolutional layers have different numbers of neurons. We show in Table 1 the comparison of PerHeFed with other state-of-the-art methods in terms of support for local model and shared model heterogeneity, each of which is subdivided into inter-layer heterogeneity and intra-layer heterogeneity.

**Table 1:** Comparison of the state-of-the-art methods in terms of support for different heterogeneous model scenarios (‘ ’, ‘ ’, ‘×’ indicates applicable, semi-applicable and not applicable, respectively)

	Local models heterogeneity		Shared models heterogeneity	
	Inter-layer	Inter-layer	Inter-layer	Inter-layer
FedAvg [4]	×	×	×	×
rHermes [19]	×	×	×	
FLOP [20]	×	×		×
HeteroFL [21]	×			
HFL [22]	×			
PerHeFed				

^*^ FLOP only considers the heterogeneity case with fully-connected layers removed, which is considered as semi-applicable

### Challenges and Our Solutions

The main challenge of our work is that there is no way to intervene in how clients design their local models and select their shared sub-networks, PerHeFed need to be flexible to convolutional networks of various structures, even different CNN models. To address the above challenge, we employ a novel aggregation method that allows heterogeneous networks to be aggregated. The details are presented in Section 3.2.

The main idea of PerHeFed is that, instead of collaboratively training models with the uniform structure in conventional FL frameworks, each device can design heterogeneous local models and the shared sub-models according to their own situations of different data, hardware resources, or other strategies previous discussed. Only the parameters of the selected partial network will be transmitted between devices and the server, which can significantly reduce the communication cost due to the compact size of the sub-models. We proposed a novel weighted aggregation for heterogeneous sub-models. In the initialization phase, parameter server generates a fit-all global model based on the structure details of all sub-models. Then the mapping relations between each sub-model and the global model are established. During the FL processes, the parameter server aggregates the sub-models based on the mapping relations, and compresses the updated global model into the same structure of each sub-model separately and sent to the clients.

We conduct experiments in different heterogeneous scenarios comparing the state-of-the-art FL frameworks (Hermes [19] and FLOP [20], etc. ) relevant to our work, using both inference accuracy and communication cost as evaluation metrics. The results are presented in Section 4.2. Our work demonstrates the feasibility of simple heterogeneous FL and cross-model hybrid FL by implementing simulation experiments. For the consideration of heterogeneous data, we also set up the non-IID data sets to simulate the scenario of personalized data. Experiments show that, compared to FLOP, in non-IID data sets our method compress half of the transmitted sub-model parameters with only a 4.38% drop in accuracy on SVHN dataset. On CIFAR-10, PerHeFed even achieves a 0.3% improvement in accuracy. Although our experiments only involve CNN, which have proven themselves in many application fields, we believe that PerHeFed is just as applicable to other types of neural networks.

### Summary of Contributions

In this work, we propose a general framework of personalized federated learning (PerHeFed), which can simultaneously (i) guarantee that the clients have full control over their local models and select different shared sub-models driven by their actual needs (ii) reduce communication cost, (iii) reduce the space of privacy leakage and model attacks.

**Fig. 1:**
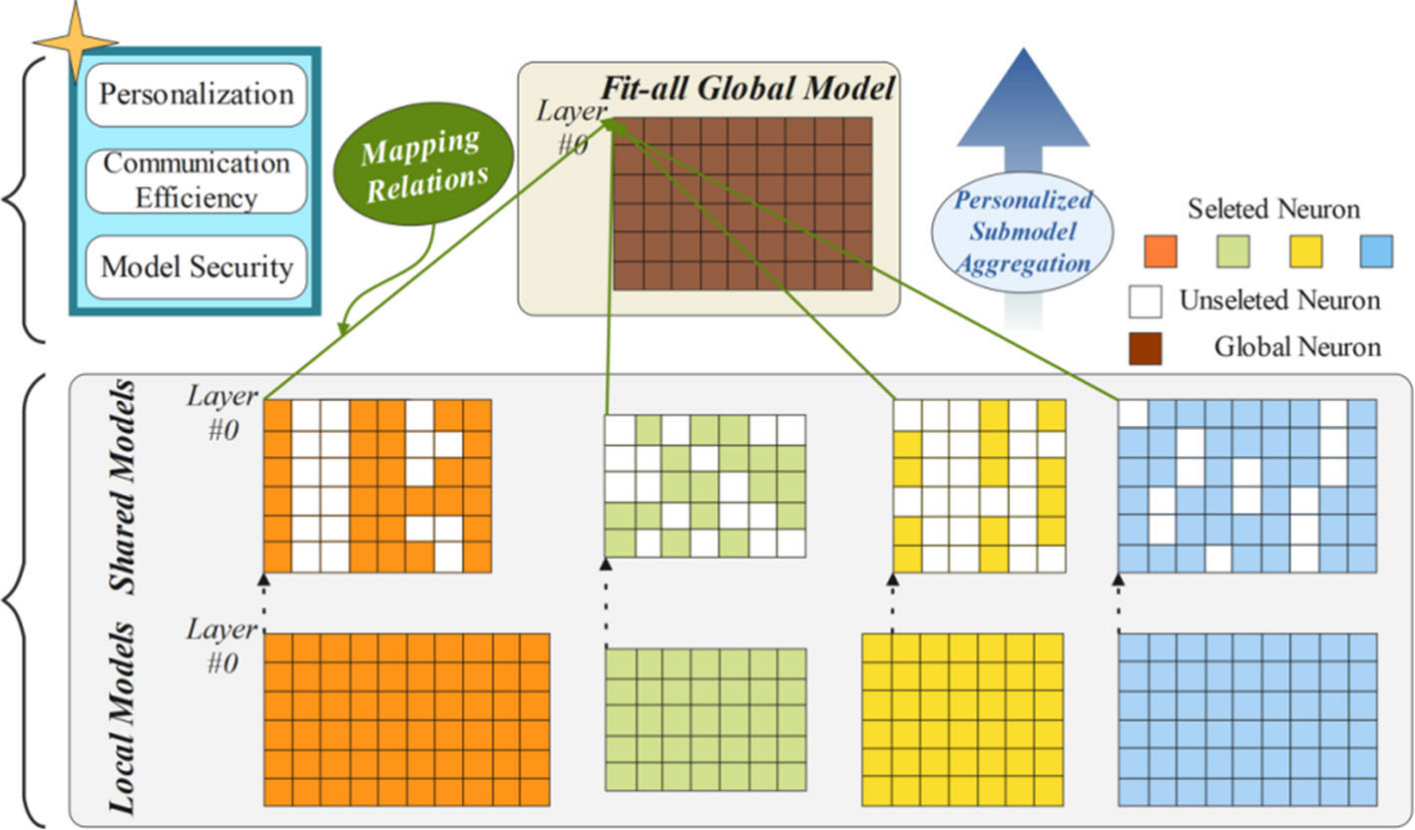
The Overview of PerHeFed

To the best of our knowledge, PerHeFed is the first general personalized FL framework for heterogeneous convolutional networks that highly achieves personalization, while improving the efficiency of communication without sacrificing the model inference accuracy. PerHeFed contributes novel techniques that address the limitations in FL under personalization settings. As shown in Figure 1, in PerHeFed, clients with different requirements no longer need to compromise for collaborative FL, but can design heterogeneous local models and freely choose their shared sub-models. Taking Layer #0 as an example, the structure of local models of each device is heterogeneous. During the parameter exchange process, devices can optionally upload the masked parameters matrices, further increasing the model heterogeneity. For the server side, it is only necessary to initialize a global model that can easily fit all heterogeneous the sub-models and the mapping relations to enable the subsequent personalized sub-model aggregation process. As in Figure 1, aggregation of heterogeneous parameter matrices relies on mapping relations. They all take index #0 as the starting point of mapping. We believe that our work represents a significant step towards applying FL to personalization scenarios requiring heterogeneous models.

## Related work

### Federated learning

FL was design to carry out high-efficiency machine learning among multiple parties or devices under the premise of ensuring the security of terminal data. FL usually consists of a parameter server (or without [21]) and several local devices. Each device uses its own data to train a local model, and the parameter server integrates all the local models into a global one. FedAvg [4] is a classic FL algorithm. It divides the training process into multiple rounds. In each round, the parameter server arbitrarily chooses several models from all devices to averagely aggregate and update the global model. The selected devices download the updated model from the parameter server, overwrite its local model parameters, and proceed to the next round of training; while the unselected devices continue to the next epoch on their original local models. During the entire learning process, the devices only communicate with the parameter server, and cannot obtain any information about the rest of the devices except for the global model that are jointly maintained, which guarantees the confidentiality of private data.

### Personalized federated learning

However, conventional FL only roughly aggregates various models, and all devices obtain the same global model, ignoring that in the context of the IoT, each device is different, both in terms of hardware resources and local data distribution. Researchers summarize the challenges FL faced in practical applications as follows [22]:

1. **System heterogeneity** such as storage, computing and communication capabilities;
2. **Data heterogeneity** like non-IID local data;
3. **Model heterogeneity**, where clients need different model structure according to their application scenarios.

To tackle these heterogeneity challenges, it is effective to perform personalization in device, data and model levels to attain well-trained personalized model for each device. Because of its promising application scenarios (such as IoT based personalized smart healthcare [23, 24], smart home services and applications, and location-aware recommendation services [25–27]), personalized learning has recently attracted great attention [28, 29]. Among them, we focus on a general personalized FL solutions to the problem of model heterogeneity.

### FL of heterogeneous models

In conventional FL, all devices including parameter servers are restricted by the unified model structure. Moreover, in the process of interaction, the complete model parameters are transmitted, which is not only unfavorable for applications in the IoT environment, but also increases the communication cost and the risk of privacy leakage. Heterogeneous models refer to models with different structures (including the number of functional layers, the structure within a layer and the combination order of different functional layers, etc.). At present, the FL of heterogeneous models has attracted the attention of some researchers. Diao [30] proposed an algorithm called HeteroFL. HeteroFL divides the domain of the global model so that each heterogeneous device model is responsible for updating partial global model. However, HeteroFL divides the global model parameter matrices from the origin. For the situation where the global model is far more complicated than the local model, some neurons of the global model cannot be updated during the whole learning process, which affects the model performance. HFL [31], is a step forward than HeteroFL. The parameter server partitions the domain that each device is responsible for as needed, so that the global model parameters can be evenly updated. At the same time, HFL also supports the fusion and compression of convolutional layer with different convolutional kernel sizes. Nevertheless, the algorithms mentioned above only discuss neural networks with the same number of convolutional layers, and cannot easily extend to different CNN models. In addition, FL algorithms of heterogeneous models with different depths have gradually emerged. Yang [20] and Collins [9] proposed to reserve the functional layers, like decision layers that are more relevant to personalization, and conducted FL of heterogeneous models by only fusing the layers before the decision layers. Furthermore, Arivazhagan [32] divided the model into basic and personalized sub-models, breaking through the previous limitation that the personalized layers only contained the decision-making layers, which explored the personalization performance of other functional layers. However, the above works only rigidly divide the model into two major parts, the basic and the personalized, rather than flexibly separating it. Secondly, the basic layers at different devices of these algorithms are completely the same from the neuron settings to specific parameter values, ignoring the need for fine-tuning the basic layers by local devices. Linet [33] and Lin [34] proposed to use knowledge distillation [35] to solve the model heterogeneity problem. This requires additional data sets (from public data sets or clients’ private data), which is against our original setting that the parameter server do not contain any data.

## Method

To tackle the challenges of model heterogeneity caused by personalized requirements, we propose a general framework for personalized FL of heterogeneous models. In PerHeFed, the devices can autonomously select the local models and the shared sub-models which are exchanged when communicating with the parameter server. The parameter server is responsible for the weighted aggregation of these partial networks. Since only the part of model parameters need to transmit in PerHeFed, it not only lowers communication overhead but also reduces the risk of privacy leakage. The algorithm is designed to be agnostic to any specific networks. Different networks and tasks can use it to obtain suitable well-trained personalized models. Without loss of generality, the model architectures in this paper are based on convolutional neural networks (CNNs). Although there are many variations of the CNN architecture, a CNN for image classification tasks is typically composed of two basic components [20]: a feature extractor and classifier. The feature extractor includes several convolutional layers followed by max-pooling and an activation function, while the classifier usually consists of fully connected layers. In the task of image classification, the CNNs extract the low-level features, such as straight edge, color, texture, and then generate a more abstract concept, to achieve classification and recognition. The convolution kernels (also called neurons or filters) can be regarded as a feature recognizer, which extract features from the input image to obtain the feature maps. Compared with other functional layers such as the fully-connected layers that belongs to the category of decision-making, the convolutional layers are considered to be less relevant to the personalized data. Motivated by this observation, we believe it is reasonable to choose the shared models from the convolutional layers instead of other functional layers. The intuition of our work is that even though the convolutional networks chosen by the clients are different, the function of their convolutional layers is the same, i.e., extracting image features, and therefore the parameters of these convolutional layers are aggregable, which has been verified in our experiments.

### Overview

**Fig. 2:**
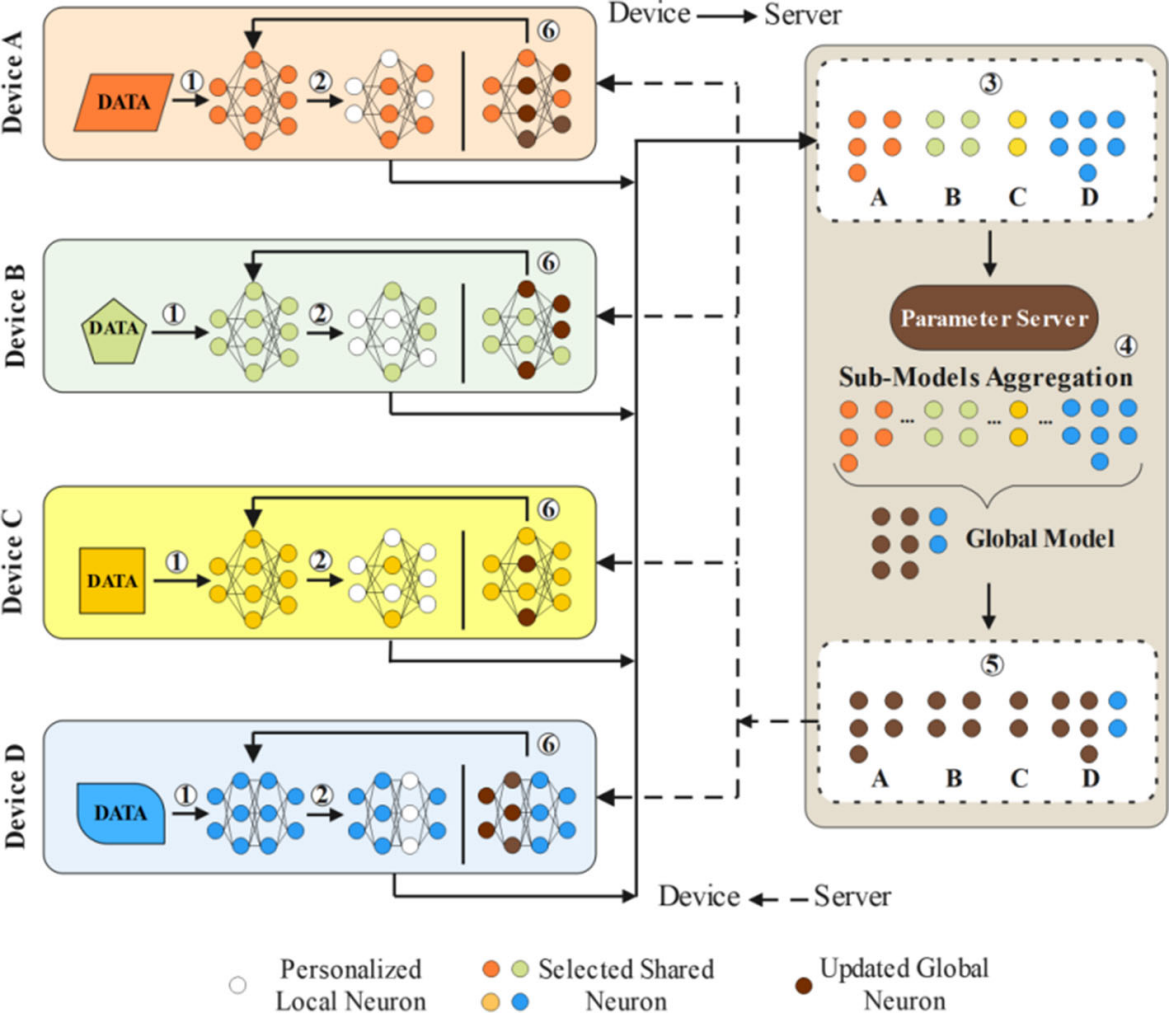
The architecture of PerHeFed framework

Figure 2 illustrates the architecture of the proposed PerHeFed framework, which involves two major parts: each clients training their local models (left part of Figure 2). In particular, at the beginning, each device initializes their local model structure according to its requirements and selects the partial (or whole) network as shared model. The sub-models can be the combinations of several convolutional layers from the original networks, the sparse versions of the original models, or even the integration of these two types. Sub-models are transferred to the parameter server. The server performs parameter aggregation based on the mapping relationship(the right part of Figure 2). The aggregated global model is likewise compressed to the parameter structures of each device model through mapping relationships, which are used to update local models.

#### Notations

Before diving into the details of PerHeFed, we first define the following notations used in our work. Suppose there are devices N={1,2,...,n} participating in FL, and each device has its own private data *D* = {*D*_1_,*D*_2_,...,*D*_*n*_} and trains its local model *M* = {*M*_1_,*M*_2_,...,*M*_*n*_} independently. Device i selects its shared model ${M_{i}^{s}}$, and the remaining unselected part of the models is considered as the private model ${M_{i}^{u}}$. The shared model ${M_{i}^{s}}$ consists of *l*_*i*_ convolutional layers, that is ${M_{i}^{s}}=\{L_{i1},L_{i2},...L_(il_{i} ) \}$. The parameter server maintains the global model *M*_*gthrough*_ the personalized sub-model aggregation of shared models from devices. We summarize the proposed PerHeFed in Algorithm 1 in Appendix A. In the initialization phase, device i sends the structure information of the selected shared model ${M_{i}^{s}}$ to the parameter server. The parameter server generates the mapping relation R accordingly. From the perspective of device i and parameter server, the steps of the entire training process are as follows:

- **Step 1:** Device i trains the local model *M*_*i*_ for T rounds based on its local data set *D*_*i*_;
- **Step 2:** After T rounds of local learning, device i suspends training and prunes the current local model to obtain shared sub-model ${M_{i}^{s}}$ based on its strategy;
- **Step 3:** The selected sub-model ${M_{i}^{s}}$ is uploaded to the parameter server;
- **Step 4:** The parameter server selects n×C among the N devices to accept their shared models and calculate the current one-time weight matrix W according to the all the mapping relation Rs. Then server conducts personalized sub-model aggregation method to aggregates the selected sub-models with W and Rs. The aggregated model will overwrite the current global model *M*_*g*_ to become the new global model $M_{g}^{\prime }$;
- **Step 5:** The new global model $M_{g}^{\prime }$ is pruned by the server before being sent back to the devices i according to the corresponding mapping relation *R*_*i*_;
- **Step 6:** After receiving the model ${{M_{i}^{s}}}^{\prime }$from server, device i combines it with its local private model ${M_{i}^{u}}$ to form a new local learning model, and starts the next epoch.

Repeat steps 2-6 until the end of the FL, all devices will obtain different trained models.

### The PerHeFed framework

In PerHeFed, there are no limitations on how clients design their local models and select the shared sub-models, so the uploaded sub-models are highly heterogeneous. The main challenge of our work is to aggregate these heterogeneous personalized sub-models and perform FL.

#### Fit-all global model

The parameter server in PerHeFed has little knowledge about the sub-model selection, so a global model that can cope with heterogeneous shared models is needed. To address this problem, we propose a fit-all global model approach. In the initialization phase, the parameter server generates the global model *M*_*g*_ based on the shared model information uploaded by the clients. The global model *M*_*g*_ consists of *l*_*g*_ = *max*(*l*_1_,*l*_2_,⋯*l*_*n*_) convolutional layers, where each layer *L*_*gis*_ the maximum size of all the corresponding shared layers in each dimension. Each uploaded shared model can be regarded as a sub-model of the global model. Based on the observations, we found that the heterogeneity of the model is mainly divided into two types: inter-layer heterogeneity and intra-layer heterogeneity. Therefore, personalized shared models can be aggregated from these two dimensions.

#### Mapping relations

**Fig. 3:**
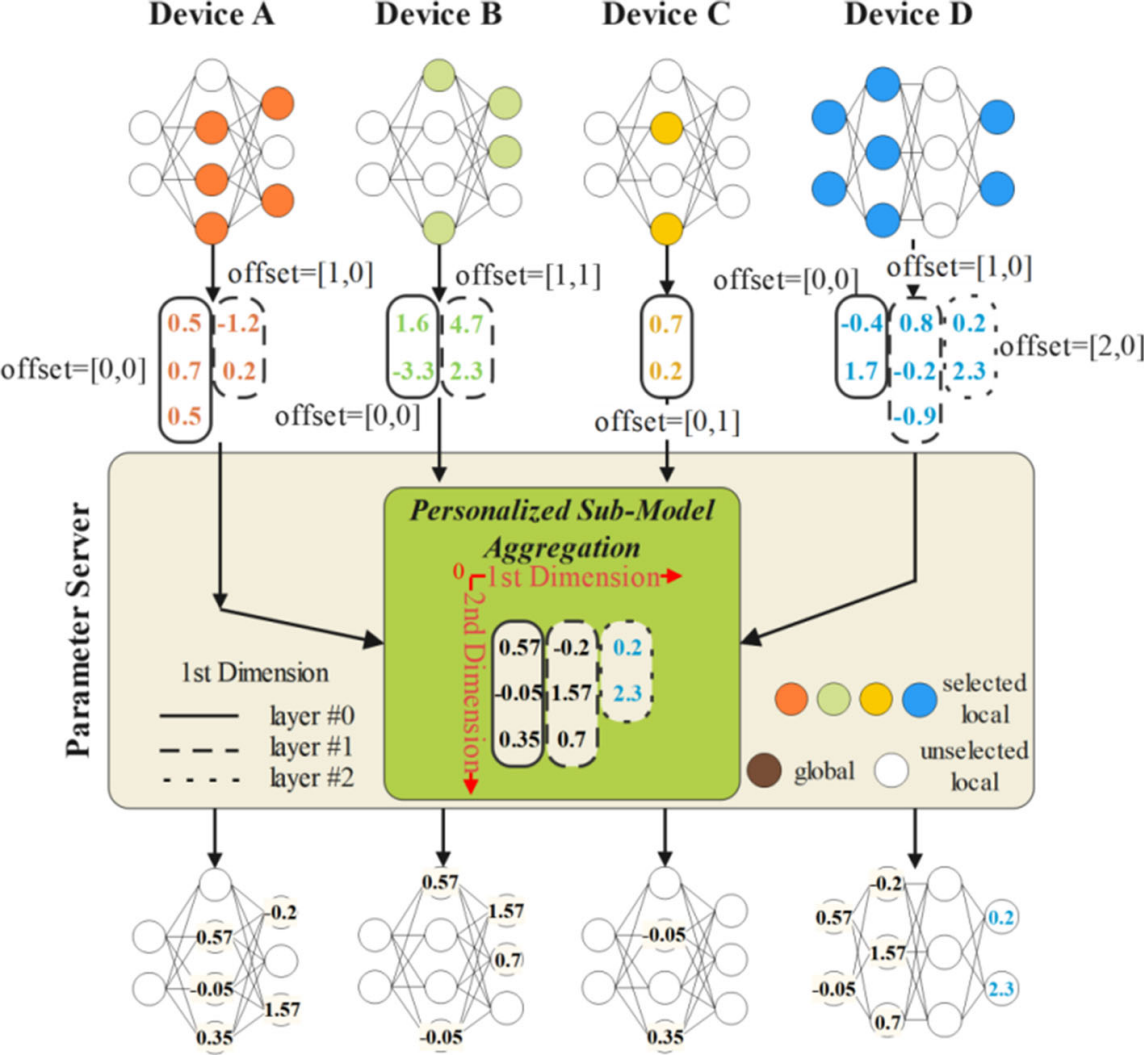
Inter-layer Aggregation (To simplify the expression, a single number is used instead of the actual 3-dimensional parameter matrix to show the aggregation algorithm details. Offset = [*a*,*b*] means the 1st dimension offset is a and the 2nd dimension offset is b. Any operations in the figure are matrix operations)

As discussed above, the selected sub-models are essentially the sets of sparse/dense parameter matrices of multiple convolutional layers, where they may from different locations and different numbers of convolutional layers. Figure 3 illustrates several examples of heterogeneous sub-models. Because of the diversity of the sub-models, we need a general mapping method that is robust to all types of sub-models.

Since the shared sub-models chosen by each client may be heterogeneous and only consist of a few convolutional layers from the original models, it makes little sense to consider layer-to-layer connections at this point. In PerHeFed, the connection of the convolutional layers in the sub-model are ignored and only the parameter matrices are kept. The parameter matrices are rearranged in the order of their original positions to form a new sequence of matrices. In the new sequence, the parameter matrices at the same positions will be bonded. The parameter server is responsible for recording such mapping relations Rs between the sub-models and the global mode. The mapping relations Rs are the sets of 2-dimensional offset matrices that can be preset in the configuration file or generated by the parameter server in the following way. And the 1st dimension describe parameters of which shared layers of sub-models are aggregated to update which global layers. Simply, in PerHeFed, the 1st dimensions of the offset matrices are generated by the number of sub-model layers. That is, the 1st dimension of the offset matrix of an n-layer sub-model is [0,1,2,...*n*], where the index I and the value K indicates the *L*_*sI*_ of the sub-model corresponds to the *L*_*gK*_ of the global model. This is based on our intuition that although the same layers of the sub-models (taking *L*_*s*0_ as an example) may be from different locations of different local models, it is the shallowest layers of the sub-models, so it should be aggregated within shallow layers rather than with other layers of the sub-models.

**Fig. 4:**
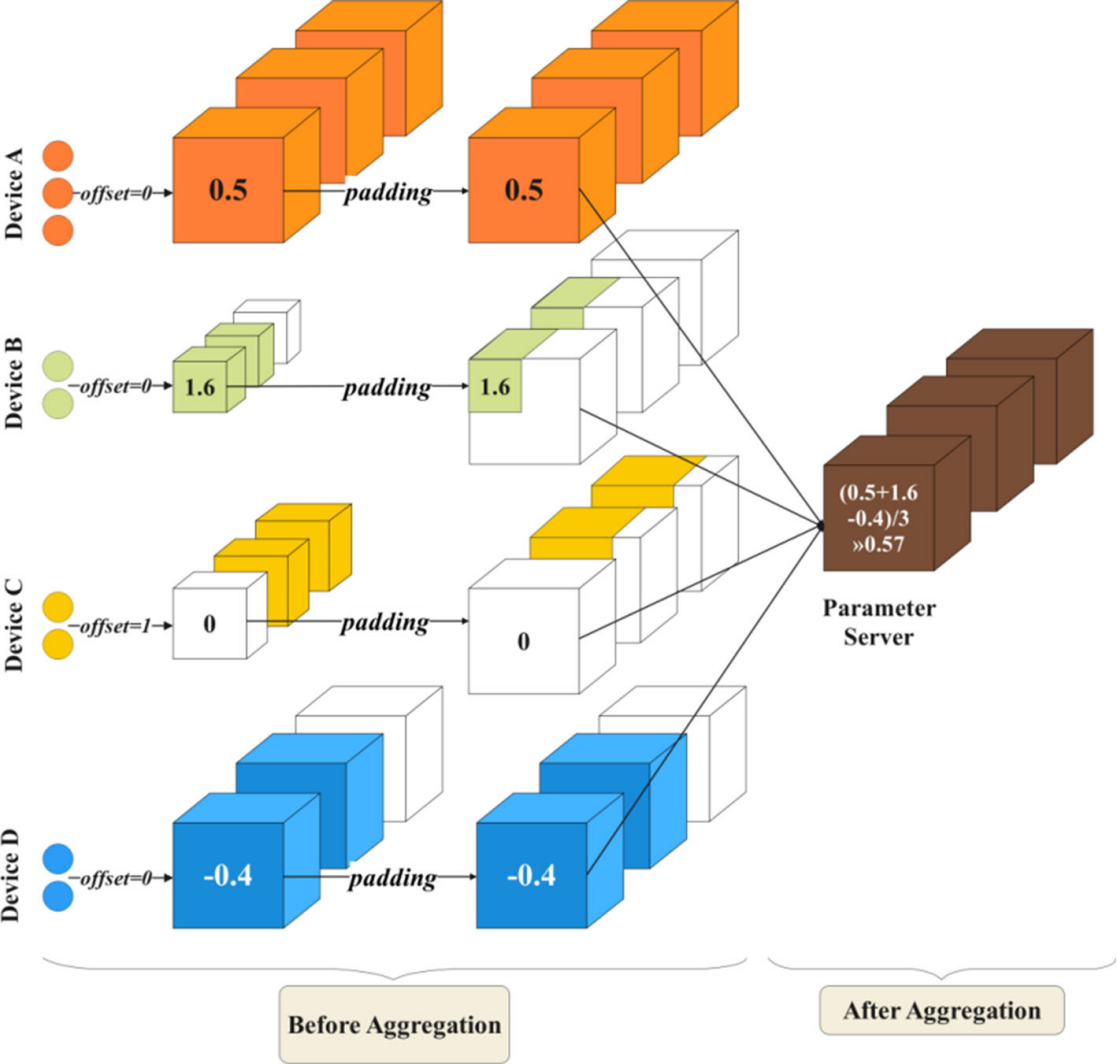
Intra-layer aggregation for the layer #0 of each sub-models in Figure 3 (Only the 2nd dimension of the offset matrix is shown here. To simplify the expression, a single number is used instead of the actual 3-dimensional parameter matrix to show the aggregation algorithm details. Any operations in the figure are matrix operations)

The parameters of the layers to be aggregated may present shape mis-matched, so the 2nd dimension of the offset matrix is needed to determine which filters of the global layers are updated by each shared layer’s filters. Figure 3 also highlights the 2nd dimension of the offset matrix. Figure 4 is the example of intra-layer aggregation for the layer #0 of each sub-models in Figure 3. In Figure 4, offset= 0 means that the parameters of filter #0 of the shared layer updates the parameter of filter #0 of the global layer and similarly, offset= 1 means that filter #0 of the shared layer matches the filter #1 of the global layer.

#### Personalized sub-model aggregation

After determining the one-to-one mapping Rs between the sub-models and the global model, the personalized sub-model aggregation can be started. As in Figure 3 the *L*_*g*0_ are updated by the aggregation of the *L*_*s*0_ of each sub-model. It is worth noting that the global model may have some layers updated by only one client, e.g., the *L*_*g*2_ in Figure 3 is only updated by the local layer #3 (i.e., the shared layer #2) of device D. In this case, the aggregation result of this layer is the same as the shared layer.

The heterogeneity of the shared model is reflected not only in the number of selected convolutional layers, but also in the intra-layer structures. In intra-layer aggregation, the parameter matrix of each filter is aggregated according to the 2nd dimension offset. As in Figure 4, filter #0 of the global layer is updated by the aggregation of filter #0 of shared layer A, B, and D.

As for the case of filter shape mis-matched, we simply use the origin-aligned approach and pad the remain parts with zero matrices. Of course, additional offsets can also be introduced to control the filter mapping just as the condition mentioned above, but will not be further discussed here. The intra-layer aggregation focuses on the mismatched intra-layer structures. For the case where the inter-layer structures are the same and only some of the parameters are pruned (possibly using mask operations for privacy-preservation reasons), sparse matrices can be aggregated directly by matrix addition operations.

In aggregation algorithms, weight is an important component. In this work, we use the number of shared parameters (i.e., the non-zero parameters accepted by the server) as the basis for weight calculation.

After the aggregation, the server prunes the global model according to the mapping relations and sends back to the clients.

## Experiment and analysis

### Implementations details

We considered 1 parameter server and 10 devices. Some od the implementation details like datasets, model architectures and baselines are presented in Appendix A. We set up two experimental scenarios:

**Simple heterogeneous scenario:** in order to simulate heterogeneous scenario, we randomly select convolutional layers from the model to reconstruct the shared sub-model according to a certain ratio *C*_*Inter*_ and introduce the ratio *C*_*Intra*_ to control the number of retained filters in selected layers.
**Cross-model hybrid scenario:** we mix two different convolutional models (ResNet34 and DenseNet121) to simulate the cross-model scenario. In this scenario, the total number of the two models is 10. We set *C*_*Intra*_= 1.0 and only vary the value of *C*_*Inter*_ to observe the model performance.

### Results

#### Communication costs

**Fig. 5:**
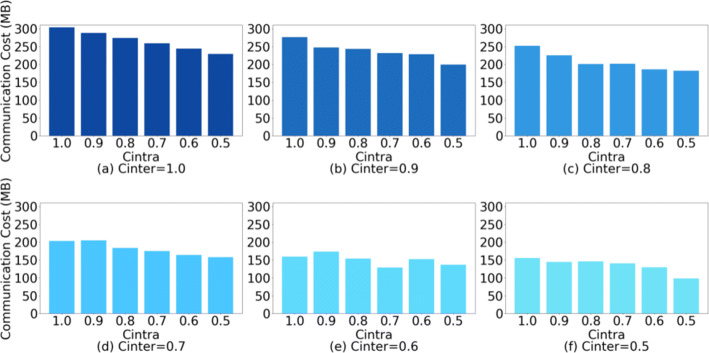
Communication cost of ResNet34 under different *C*_*Inter*_ and *C*_*Intra*_ combinations

**Fig. 6:**
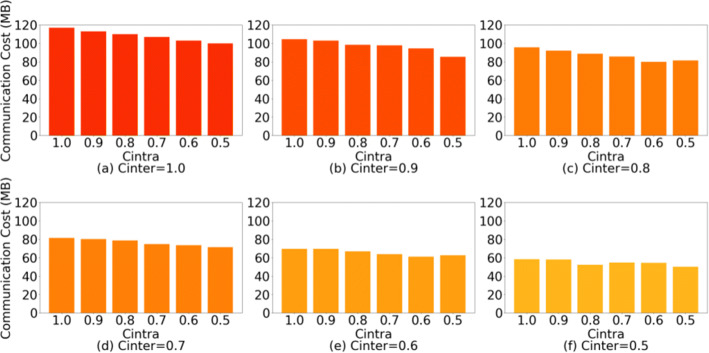
Communication cost of DenseNet121 under different *C*_*Inter*_ and *C*_*Intra*_ combinations)

We show the communication cost of our approach in the above two scenarios. We use the number of sub-model parameters to measure the actual communication cost. Although *C*_*Inter*_ and *C*_*Intra*_ control the number of retained layers and intra-layer parameters, which specific layers and filters are selected in the experiments is still random, and we conduct multiple sets of experiments to mitigate experimental data fluctuations due to randomness. Figure 5 shows the communication cost of ResNet34 (DenseNet121 case is shown in Figure 6) under different *C*_*Inter*_ and *C*_*Intra*_ combinations. In the case of *C*_*Inter*_ = 0.5 and *C*_*Intra*_= 0.5, the communication cost of ResNet34 in our method is 0.33 times (0.42 times in the DenseNet121) that of the conventional FL (same in FedAVG).

#### Inference accuracy

**Simple heterogeneous scenario:** Table 2 shows the inference accuracy when *C*_*Intra*_= 1.0. The shown results are the average accuracy of ResNet34 and DenseNet121. We cannot conclude a regular effect of *C*_*Inter*_ on the inference accuracy from Table 2. Combining Figures 5 and 6, it can be observed that *C*_*Inter*_ has a greater impact on communication costs than *C*_*Intra*_, while at the same time, when *C*_*Inter*_ ∈ [0.5,0.6,0.7,0.8,0.9], it has little effect on the accuracy. Although compared to the case of *C*_*Inter*_= 1.0, the accuracy of *C*_*Inter*_ ∈ [0.5,0.6,0.7,0.8,0.9] is still decreased ≈ 8.263*%*.

**Table 2:** Testing accuracy for PerHeFed(when *C*_*intra*_= 1)

	0.5	0.6	0.7	0.8	0.9	1.0
SVHN	82.5%	82.2%	82.3%	82.6%	82.4%	90.7%
Cifar10	61.2%	61.0%	61.3%	61.8%	62.9%	75.4%

To show how the accuracy varies with *C*_*Intra*_, we calculate the average of the accuracy with different *C*_*Inter*_ and show it in Table 3. Basically, it can be observed that the inference accuracy increases as the value of *C*_*Intra*_.

**Table 3:** Average of testing accuracy for PerHeFed(when *C*_*Inter*_ ∈ [0.5,0.6,0.7,0.8,0.9,1.0])

	0.5	0.6	0.7	0.8	0.9	1.0
SVHN	82.5%	82.2%	82.3%	82.6%	82.4%	90.7%
Cifar10	61.2%	61.0%	61.3%	61.8%	62.9%	75.4%

**Fig. 7:**
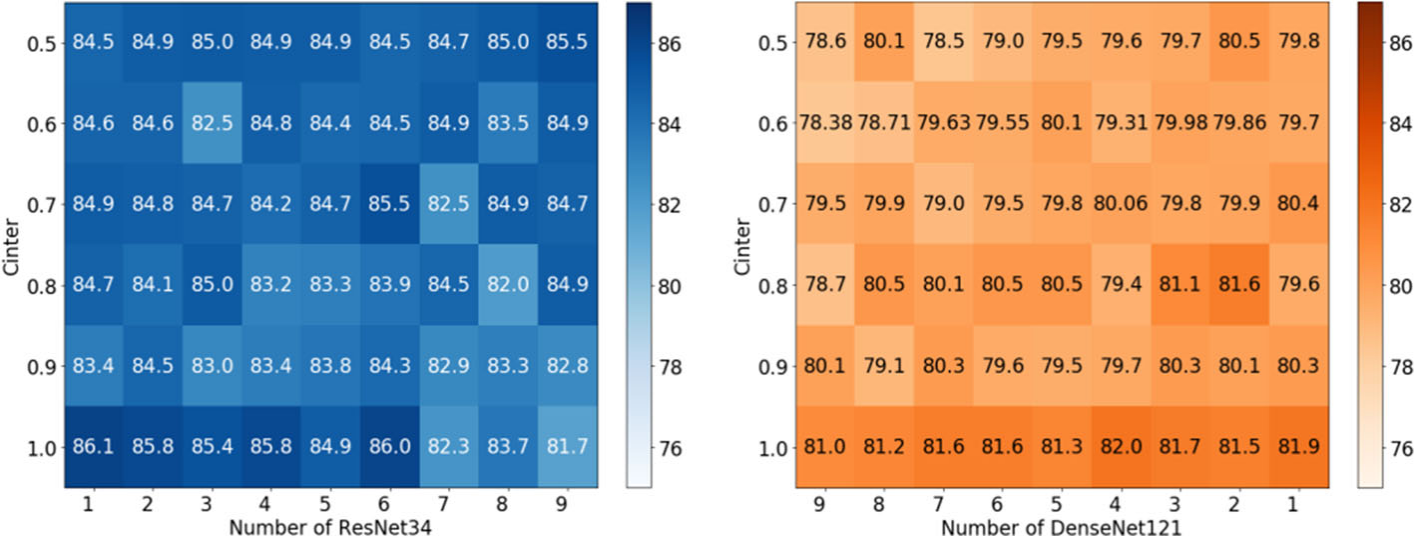
Testing accuracy of SVHN dataset in cross-model hybrid scenario. Subfigure (a) and (b) come from the same batch of experiments. I.e., in the experiment where ten models contain 1 ResNet34 and 9 DenseNet121 models. The inference accuracy of *num*_*ResNet*34_ = 1 in subfigure (a) and *num*_*DenseNet*121_= 9 in (b) show the average performance of the ResNet34 and DenseNet121 models, respectively

**Fig. 8:**
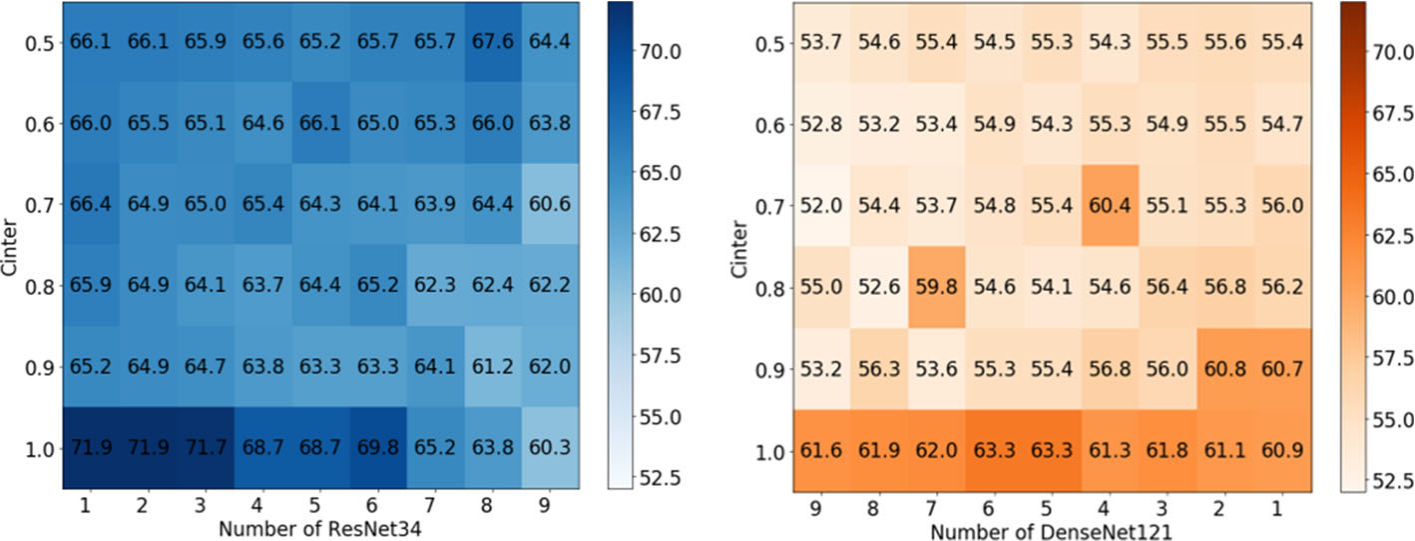
Testing accuracy of CIFAR-10 dataset in cross-model hybrid scenario. Subfigure (a) and (b) come from the same batch of experiments. I.e., in the experiment where ten models contain 1 ResNet34 and 9 DenseNet121 models. The inference accuracy of *num*_*ResNet*34_ = 1 in subfigure (a) and *num*_*DenseNet*121_ = 9 in (b) show the average performance of the ResNet34 and DenseNet121 models, respectively

**Cross-model hybrid scenario:** The accuracy of the confounding patterns under the SVHN datasets are shown in Figure 7 (the results of CIFAR-10 are shown in Figure 8). Although the shown data fail to reveal a clearer pattern, in general, it can be observed that the accuracy of the mixed models is similar to that of the previous simple heterogeneous scenario. Only 7.6% (83.1%-90.7% on SVHN) and 10.5% (64.9%-75.4% on CIFAR-10) accuracy was sacrificed when *C*_*Inter*_= 1.0. We believe that the noises caused by cross-models affects the accuracy, as can be seen from the darker color at the ends than in the middle region of the figures. We will continue to investigate how to reduce the adverse effects on the global performance brought by the widely different shared models in future work. Overall, our work demonstrates the feasibility of cross-model FL, where the convolutional layer parameters from different positions of different models are fusible.

#### Baselines

To verify the inference accuracy of our method, we chose the well-known algorithm FedAvg, the state-of-the-art method FLOP and Hermes as our baseline. Since the baseline methods only applicable to simple heterogeneous scenario, the following experiments are all based on the simple heterogeneous scenario.

**Fig. 9:**
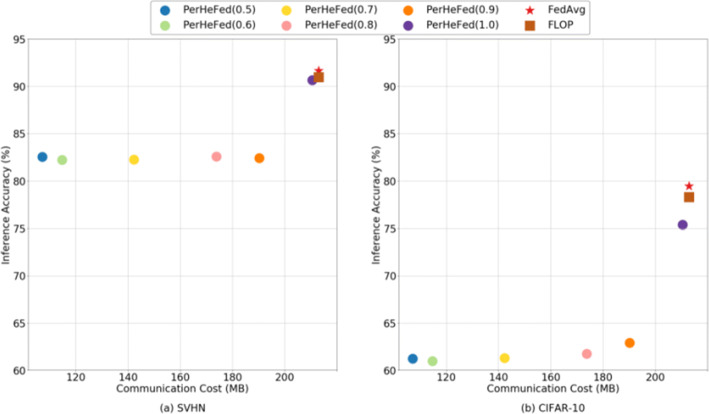
Comparison between PerHeFed and baselines (FedAvg and FLOP) in inference accuracy-communication cost space

**Fig. 10:**
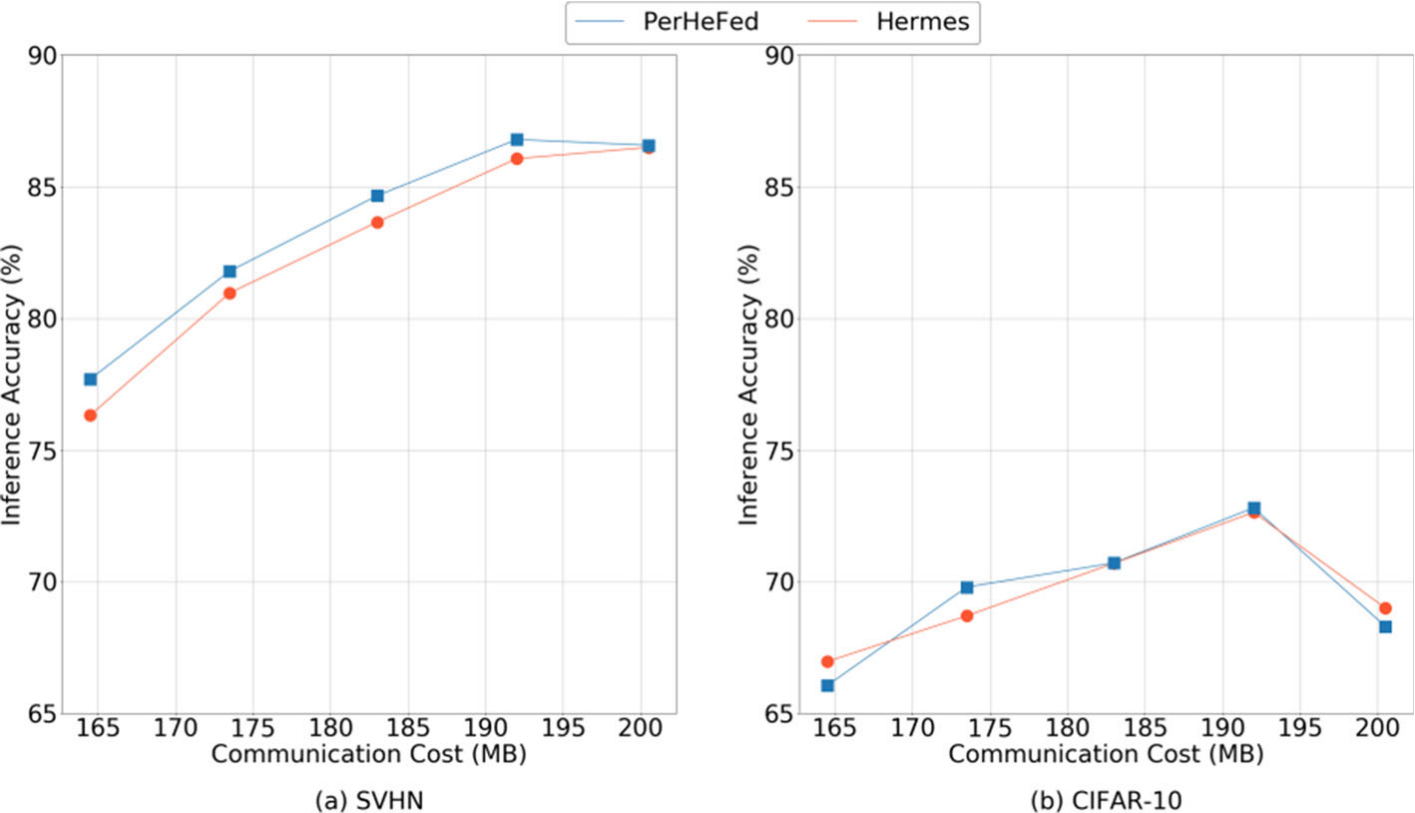
Comparison between PerHeFed and Hermes in inference accuracy-communication cost space

**FedAvg and FLOP:** To compare with Fedavg and FLOP, we set *C*_*I*_*ntra*= 1.0 and change only the value of *C*_*Inter*_. Even though our method cannot always outperform FedAvg and FLOP in both inference accuracy and communication cost, it offers the best trade-off between inference accuracy and communication cost. PerHeFed is a general heterogeneous FL framework suitable for a variety of clients with personalized needs. Clients who need high accuracy models can select high computing complexity shared sub-models (as in the case simulated in the Figure 9 when *C*_*Inter*_= 1.0), in this case, our method achieves similar accuracy to FedAvg and FLOP; while clients with limited bandwidth or concerned about the security of shared models can also prune to obtain streamlined sub-models, accuracy is sacrificed to some extent, as the case in Figure 9 when *C*_*Inter*_= 0.5, sacrificing 8.5% (on SVHN) /17.1% (on CIFAR-10, which we believe is partly due to model overfitting) of accuracy in exchange for a 0.5-fold reduction in model parameters of sub-models compared with FLOP.
**Hermes:** To compare with Hermes, we set *C*_*Inter*_= 1.0 and change only the value of *C*_*Intra*_. Figure 10 show that, in general, our method (blue lines) achieves slightly high accuracy than Hermes (orange lines) and with the same value of *C*_*Intra*_, the communication cost of the two methods is the same.

#### Experiments on extreme non-IID data

**Fig. 11:**
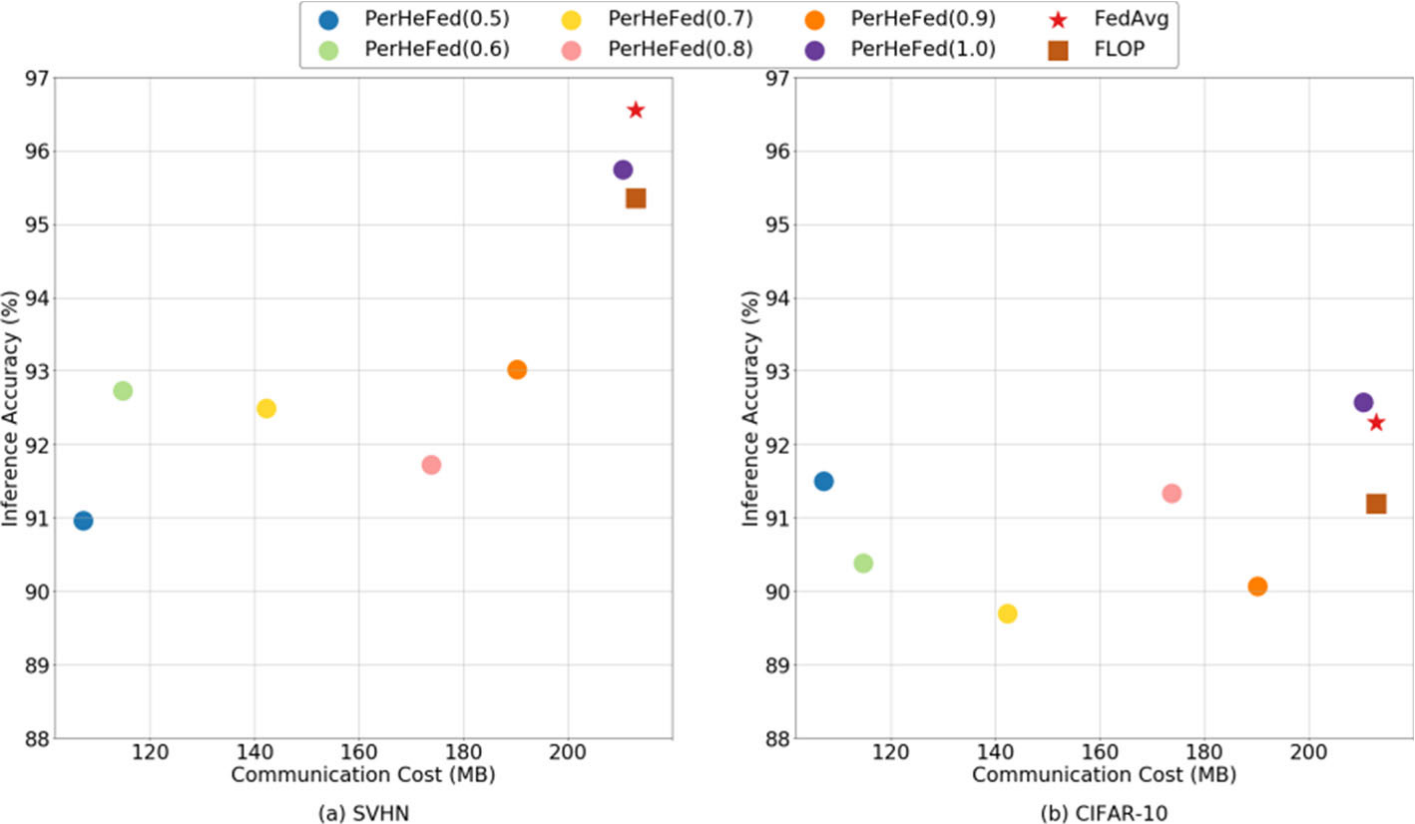
Comparison between PerHeFed and baselines (FedAvg and FLOP) in inference accuracy-communication cost space on non-IID data sets

Although the data distribution of previous experiments is likely to be non-IID, to further explore the performance of PerHeFed on non-IID datasets, we adopt the more extreme non-IID configure described in Appendix A. Comparing Figures 9 and 11, it can be seen that the accuracy gaps between our method and the baseline methods are reduced on the non-IID data sets. Our method reduces the communication cost to one-half of the original cost on SVHN, with only a 4.38% drop in accuracy. On CIFAR-10, PerHeFed even achieves a 0.3% improvement in accuracy.

**Fig. 12:**
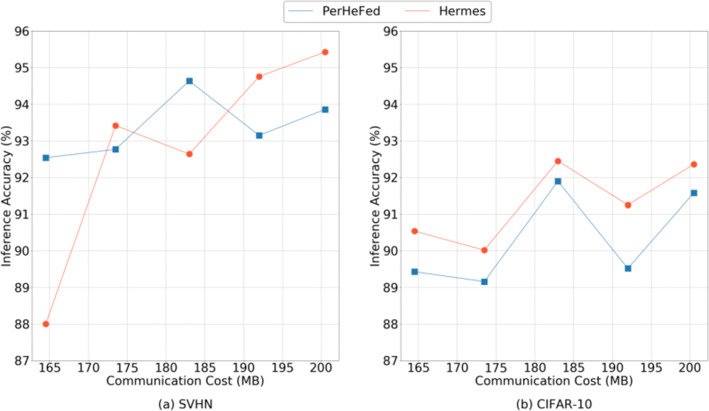
Comparison between PerHeFed and Hermes in inference accuracy-communication cost space on non-IID data sets

Figure 12 shows the comparison with Hermes in extreme non-IID setting. In general, although the inference accuracy of our method (blue lines) is slightly lower than Hermes (orange lines) and with the same value of *C*_*Intra*_, PerHeFed can provide clients with more control over the selection of component parts of shared sub-models.

#### Discussion

PerHeFed is a general personalized FL framework for heterogeneous models, aiming to supports all personalized requirements of devices regarding model heterogeneity. In PerHeFed, a trade-off between model heterogeneity and accuracy can be observed. The aggregation of the parameters from different convolutional layers can add some noises to the global model to some extent (see Table 2), thus affecting the performance of the local model. The greater the variability of the shared models, the more likely it is to affect the model accuracy (see Figures 7 and 8).

However, from the above experimental results, the sacrifice of accuracy seems to be negligible compared to the positive effects of heterogeneous models such as saving communication costs and reducing the space of privacy leakage and model attacks. And we believe that we can use methods such as model fine-tuning, data augmentation, etc. to improve the inference accuracy. Again, this trade-off needs to be considered before conducting the FL processes. And as a general framework for FL of heterogeneous model, PerHeFed can support both combinations of heterogeneous models and the widely accepted version of homogeneous models as well.

## Conclusion

Conventional FL requires all local devices to train network models with the same structure, which is difficult to satisfy in the personalization application scenarios. Secondly, previous studies have shown that the transmission of complete model information in conventional FL has the risk of privacy leakage. In order to tackle the challenges mentioned above, we propose a general framework for personalized federated learning (PerHeFed). We believe PerHeFed is the first general personalized federated learning framework for heterogeneous convolutional networks, even cross different networks, addressing model structure unity in conventional FL.

PerHeFed can meet the needs of different federated devices to train heterogeneous model by integrating their partial models. The participating devices can select the shared layers based on their own wishes, and only exchange them when communicating with the parameter server. Since only the partial model parameters are needs to exchange in PerHeFed, it not only lowers communication overhead but also reduces the risk of privacy leakage. Our method is designed to be agnostic to any specific networks. Different networks and tasks can use it to obtain suitable well-trained models. In non-IID data sets our method compress half of the shared sub-model parameters with only a 4.38% drop in accuracy on SVHN dataset. On CIFAR-10, PerHeFed even achieves a 0.3% improvement in accuracy.

In the future, we plan to study how to reduce the adverse effects on the global performance brought by the widely different shared models. And it is worthwhile to explore the boundaries of heterogeneous models for FL, and regulate the behavior of clients participating in FL to avoid model poisoning of the global model. In addition, we will study how our approach can be implemented in other neural networks such as RNNs.

## Figures and Tables

**Algorithm 1:**
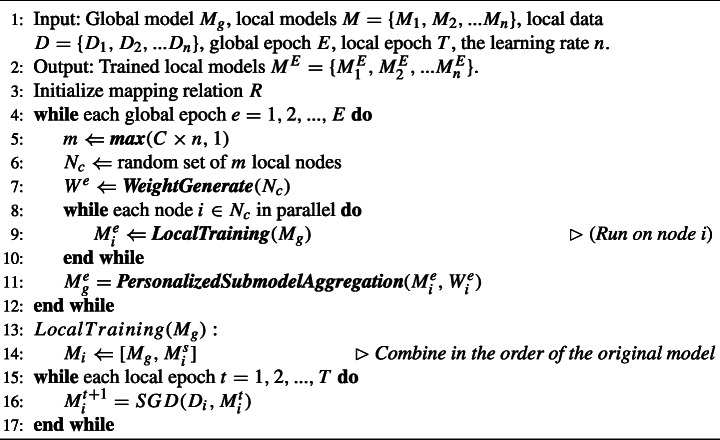
PreHeFed(Server).

## Data Availability

The data supporting the findings of this study are public data and are available online.

## Appendix A

We provide pseudo-code of Algorithm 1, the employed experimental settings and supplementary experimental results.

### A.2 Supplementary Implementation Details

#### A.2.1 Datasets

##### CIFAR-10 [36]

is an image classification data set with 10 labeled classes. It contains 50,000 training samples and 10,000 testing samples.

##### SVHN [37]

is a real-world image data set that can be regarded as similar to the MNIST (e.g., the images are of small cropped digits). SVHN is obtained from the house number in the Google Street View image. It contains 73257 training samples and 26032 testing samples.

We shuffle the training set and divide it evenly into 10 subsets, one of which is taken randomly by each client. Because the data are disordered then split, the data distribution of each subset is likely to be non-IID. The test set in SVHN has 20% of the total data volume, and the test set in CIFAR-10 is the complete original test set. To further test the performance of PerHeFed on non-IID data sets, we adopt the more extreme non-IID configure as follows: we divide the data into categories, and each category is then evenly divided into two parts. Each device holds 2-class data and these two classes are varied across devices. The test data follows the same distribution as the training data.

#### A.2.2 Model Architectures

##### ResNet34 [38]

is a residual learning framework, which can effectively solve the problem of gradient disappearance/gradient explosion that often occurs during deep neural network training. It uses residual blocks to improve the performance of very deep neural networks. At present, ResNet has been widely used in many computer vision tasks.

##### DenseNet121[39]

is a network architecture where each layer is directly connected to every other layer in a feed-forward fashion (within each dense block). For each layer, the feature maps of all preceding layers are treated as separate inputs whereas its own feature maps are passed on as inputs to all subsequent layers.

#### A.2.3 Baselines

To make fair comparison, we compare PerHeFed with three baselines:

##### FedAvg [4]

is the most classical FL method. Devices communicate updated local parameters to the central server and download the aggregated global model for continuous local training.

##### Hermes [19]

is a communication and inference-efficient FL framework under data heterogeneity. In Hermes, each device finds a small sub-network by applying the structured pruning. The communication between the devices and the server is only about the updates of these sub-networks.

##### FLOP [20]

is an effective algorithm that shares only a partial model between the server and clients. And Extensive experiments are conducted on benchmark data and real-world healthcare tasks which show that FLOP achieves comparable or better performance than FedAvg.

#### A.2.4 Other Implementation settings

We conducted our experiments in a pseudo-distributed setting on 6 Nvidia RTX 2080 Ti GPU. The related hyperparameter settings are shown in Table 4. In ResNet34, the learning Rate is 0.0001, and in DenseNet121, the learning Rate is 0.001.

**Table 4:** Hyperparameters

	CIFAR-10	SVHN
Global epoch	200	100
Local epoch	5	2
Batchsize	256	256
Optimization	Adam	Adam
